# Infrared thermography to assess fatigue, injury risk factors and recovery in soccer: a systematic review of original studies

**DOI:** 10.3389/fphys.2026.1835464

**Published:** 2026-06-18

**Authors:** Yehinson Barajas Ramón, Julio Calleja-González, José Luaces-Carreño, Álvaro Velarde-Sotres

**Affiliations:** 1 Facultad de Ciencias de la Salud, Universidad Europea del Atlántico, Santander, Spain; 2 Department of Physical Education and Sport, Faculty of Education and Sport, University of the Basque Country (UPV/EHU), Vitoria, Spain; 3 Universidad de La Romana, La Romana, Dominican Republic; 4 Fundación Universitaria Internacional de Colombia, Bogotá, Colombia; 5 Departamento de Salud, Universidad Internacional Iberoamericana, Campeche, Mexico; 6 Faculdade de Ciências de Saúde, Universidade Internacional do Cuanza, Cuito, Bié, Angola; 7 Departamento de Salud, Universidad Internacional Iberoamericana, Arecibo, PR, United States

**Keywords:** assessment, fatigue, infrared thermography, recovery, soccer

## Abstract

**Background:**

Recovery after a training session or match is a key factor in injury prevention and sports performance. The purpose of this systematic review was to analyze and consolidate the available scientific evidence from the main databases on the use of infrared thermography in the assessment of fatigue, injury risk factors, and recovery in soccer players.

**Methods:**

The literature search was conducted following the PRISMA guidelines and the PICOS model until June 30, 2025, in the main scientific databases (ScienceDirect, EMBASE, Web of Science (WOS), Cochrane Library, SciELO, MEDLINE/PubMed, SPORTDiscus, and Scopus). The risk of bias and methodological quality were assessed using the Cochrane Handbook guidelines and the PEDro scale.”

**Results:**

The initial literature search yielded a total of 510 records. After applying the inclusion and exclusion criteria, the final sample consisted of 20 studies, which were of high methodological quality. The results showed the effects of infrared thermography in assessing fatigue, identifying injury risk factors, and monitoring recovery processes in soccer players. The studies also systematically reported the characterization of the population, the assessment methods used, the variables analyzed, the methodological design, the main results, and the effects of the intervention.

**Conclusions:**

Infrared thermography shows promise as a valid, reliable, and non-invasive tool for assessing skin temperature, reflecting temperature changes in response to physiological processes. It allows for the analysis of structural or metabolic fatigue and thermal asymmetries. Therefore, thermography could be used to design individualized recovery protocols.

## 1 Introduction

An elite athlete accumulates a large number of sporting events throughout a season, which is defined by the number of championships and the time of rest them, resulting in a significant accumulation for both internal and external loads. Optimal integration and synergy of the components of physical preparation led to elite performance and sporting success, which are constantly evolving due to long-term systematic sport preparation (Calleja-González et al., 2023). Individual monitoring of athletes during training, competitions and post-competition periods can help determine their level of fatigue of athletes and the associated risk of injury (Oliveira et al., 2025). This contributes to the development of personalized recovery protocols (Altarriba-Bartes et al., 2021) tailored to each athlete’s specific characteristics, enabling them to meet the demands of each competition (Altarriba-Bartes et al., 2021).

The players’ recovery is important for them to improve their performance in team sports during high-density periods of competitions and tournaments, where players compete many times in a short period (Calleja-González et al., 2018), with little space for rest. In that sense, fatigue thus becomes an important variable and an early warning sign of potential injury (Soligard et al., 2016). Fatigue is a complex phenomenon with different manifestations, which has a variety of possible mechanisms (Halson, 2014) making it necessary to control it by adjusting training loads and periods of rest. For this reason, it is essential for coaches to consider and apply modifications to training session content during the 72-hour post-match intervention period (Marqués-Jiménez et al., 2022). This ensures that training loads are managed effectively and efficiently, allowing athletes to maximize recovery and performance within this timeframe (Silva et al., 2018). To identify fatigue-related issues and optimize sports performance, individual evaluation and monitoring should be conducted throughout training sessions and matches, including both pre- and post-competition assessments.

Given the need for objective and accurate evaluations to determine fatigue and injury risk, teams must rely on new assessment technologies (Daab et al., 2021) which have shown significant and positive effects on elite athletes’ recovery strategies (Altarriba-Bartes et al., 2020). Technology as an evaluation and monitoring tool has gained an important place in sports science, playing a vital role in athlete performance (Linnamo, 2023). These tools allow for real-time data collection, which supports informed decision-making, adjustment of training plans and workloads, and clear, objective, and rapid communication between the technical staff and athletes (Schelling et al., 2021).

Technologies such as OptoGait, which use photoelectric cells, provide a valid and reliable estimation (Lienhard et al., 2013) of flight time and vertical jump height. This system is used to evaluate counter jump (CMJ) performance (Markovic et al., 2004) offering key biomechanical insights into lower limb power—an important performance indicator and a factor linked to musculoskeletal injuries (Hoshikawa et al., 2013). Significant unilateral dominance or asymmetries detected during testing can indicate poor motor responses, which increase the risk of lower limb injuries, including heightened peripheral fatigue and excessive strain on the muscles’ elastic capacity (Menzel et al., 2013). On the other hand, Tensiomyography (TMG), which involves electrically stimulated contractions applied to the muscle belly surface and records the radial muscle deformation (Lohr et al., 2019) offers greater benefits compared to other surface mechanomyography (MMG) methods, which results in a greater impact on the contraction (Križaj et al., 2008). TMG is used to determine parameters related to fatigue state, muscle activation, muscle tone, and muscle balance (García-Manso et al., 2010).

Within this context, it is important to utilize functional and observational assessment tools that (Luchini et al., 2021) are both time-efficient and effective in evaluating muscle strength, flexibility, proprioception and coordination. These assessments provide an accurate picture of the musculoskeletal status and help identify potential injury risks in the athlete (Paszkewicz et al., 2013). Another important aspect to consider is the use of biomarkers (Schelling et al., 2015). Key variables have been established for evaluation, providing valuable parameters related to an athlete’s status, such as nutritional and metabolic health, hydration status, muscle condition and endurance performance (Lee et al., 2017). These biomarkers, which may vary daily based on the training sessions, can help detect and control warning signs linked to poor athletic performance, overload and risk of injury (Lee et al., 2017; Pedlar et al., 2019). Lastly, a critical component in monitoring and controlling athlete performance is the intensity and duration of training load during each session. This is typically measured using parameters such as distance covered in various zones, total distance, and running speeds (Luteberget et al., 2018). In sports performed outdoors, inertial measurement tools such as accelerometers and Global Positioning Systems (GPS) are used. Among these, Global Navigation Satellite Systems (GNSS) are some of the most commonly used methods for obtaining kinematic metrics in team sports (Malone et al., 2017).

A present topic of great relevance is the study of body temperature and its relationship with a person’s state of health. In this sense, infrared thermography (IRT) has emerged as a valid (Requena-Bueno et al., 2020), non-invasive and accurate tool for evaluating skin temperature (Hillen et al., 2020). It has gained significant popularity in sports as a protocol for detecting fatigue and possible risks of injury in the shortest possible time, using highly reliable image analysis (de Andrade Fernandes et al., 2014) through the ThermoHuman (Requena-Bueno et al., 2020), which detects changes in temperature as a response to physiological processes or pathological reactions (Korman et al., 2016) considering that physiological fatigue may result from either structural or metabolic processes. Structural fatigue increases temperature due to mechanical stress, whereas metabolic fatigue decreases temperature after exercise (Thorpe, 2021) Based on these on these findings, individualized recovery protocols can be designed and implemented for athletes (Calleja-González et al., 2018; Calleja-González et al., 2019).

It is important to determine the type of fatigue, structural or metabolic, in order to design recovery strategies involving either warm-up or cool-down protocols, depending on the physiological mechanism of fatigue (Thorpe, 2021) highlighting the importance of monitoring processes and the use of this technology. The IRT is a reliable instrument (Requena-Bueno et al., 2020) for identifying the origin of fatigue and determining recovery and injury prevention strategies (Fernández-Cuevas et al., 2015). One of its advantages is that it is a remote technique capable of assessing large regions of interest (ROIs), allowing for the calculation of average temperatures in body segments (Requena-Bueno et al., 2020). However, technical, environmental, and individual factors can influence and affect the results of these evaluations (Fernández-Cuevas et al., 2015). Therefore, it’s recommended to follow protocols (Korman et al., 2016) that minimize these factors. It is thus essential to understand the mechanisms of this tool to control and monitor fatigue and the risk of injury by designing tailored recovery strategies, which are the key to improving sports performance. To achieve this, new technologies are needed to assess injury risk and fatigue (Hader et al., 2019).

To date, and to the best of our knowledge, there are no previous Level 1A studies that demonstrate the use and efficacy of IRT variables for controlling and monitoring recovery in soccer players.

Accordingly, the purpose of this systematic review was to analyze and consolidate the available scientific evidence from the main databases on the use of infrared thermography in the assessment of fatigue, injury risk factors, and recovery in soccer players, providing specific and specialized information on the effectiveness of this technological tool. The above provides updated literature, contributing to better analysis, use, and development of new research proposals in the area.

## 2 Methods

### 2.1 Search strategies

This systematic review was focused on infrared thermography as a tool to assess fatigue, injury risk factors and recovery in soccer. The review was carried out following the structure as recommended by the Preferred Reporting Items for Systematic Review and Meta Analysis (PRISMA) guidelines (Liberati et al., 2009). It is registered in PROSPERO (ID = CRD420251030310). Taking into account the guidelines in the Cochrane manual (Higgins, 2011), the methodological issues were resolved.

**Figure 1:**
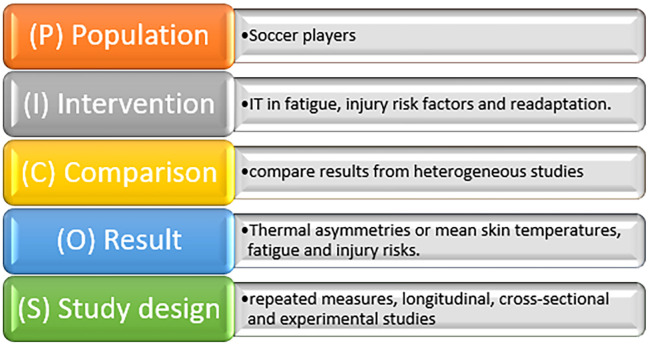
PICOS model.

The inclusion criteria were determined using the PICOS model: (P): Soccer players, (I): IRT in sports injury risk assessment, comparison (C): compare results from heterogeneous studies, outcome (O): Thermal asymmetries or mean skin temperatures, fatigue and injury risks, and finally, study design (S): repeated measures, longitudinal, cross-sectional and experimental studies. The selected articles were published in English and in JCR journals (Verhagen et al., 1998) (**Figure 1**).

In accordance with PRISMA guidelines, a systematic literature search was conducted in the ScienceDirect, EMBASE, Web of Science (WOS), Cochrane Library, SciELO, MEDLINE/PubMed, SPORTDiscus, and Scopus. The search was conducted until June 30, 2025. The search strategy combined medical subject headings (MeSH) and free-text terms related to infrared thermography assessment in soccer. The following search equation was used to find relevant articles: [“thermography” (MeSH Terms) OR “thermography” (All Fields)] OR “infrared thermography” (MeSH Terms) OR “infrared thermography” (All Fields)] AND [“recovery” (MeSH Terms) OR “recover” (All Fields)] AND [“injury” (MeSH Terms) OR “injuries” (All Fields) OR “sports injury” (MeSH Terms) OR “sports injuries” (All Fields)] AND [“fatigue” (All fields) OR “muscle fatigue” (All fields)] AND [“soccer” (MeSH Terms) OR “football” (All fields)] AND [“players” (MeSH Terms) OR “soccer players” (All fields)].

This search strategy identified all potentially relevant studies in the area of interest. In addition, a manual search of the reference lists of the included articles was performed, applying the “snowball” strategy (Greenhalgh and Peacock, 2005) in order to identify additional relevant studies. All records retrieved were checked for the detection and elimination of duplicates, as well as for the identification of possible omitted studies. Titles and abstracts were evaluated to determine their eligibility for a full-text review. The process of searching and selecting published studies was performed independently by two reviewers (Y.B.R. and A.V.-S.), and any discrepancies were resolved by consensus through discussion between both authors.

### 2.2 Inclusion and exclusion criteria

The studies included in this review provided efficacy results related to the accuracy of fatigue assessment, the identification of injury risk factors, and the analysis of recovery processes in soccer players through the use of infrared thermography. The systematic review considered only original studies, excluding systematic reviews, meta-analyses, conference abstracts, and opinion articles. Likewise, a minimum sample size of 10 participants and the use of infrared thermography as the main assessment technology were established as eligibility criteria.

The following inclusion criteria were applied to the final selected studies: (I) studies published in peer-reviewed journals; (II) original articles published in refereed journals with impact factor; (III) participants were evaluated with IRT; (IV) study population consisted of soccer players; (V) included assessment of fatigue and injury risk; (VI) conducted on athletes of any category, level of experience, degree of competition, or gender, without restrictions based on these variables; (VII) published in English. The following exclusion criteria were applied to the experimental research protocols: (I) studies with fewer than 10 participants; (II) studies that will not be conducted with soccer players; (III) abstracts, non-peer-reviewed articles and book chapters; (IV) systematic or narrative reviews.

### 2.3 Study selection

To select the items, apply the following filters and steps:

Initial filter: Articles that were not original studies, such as narrative revisions, book chapters or editorials, were excluded.

Editing of titles and summaries: Two reviewers (Y.B.R and J.L.-C) independently examined the titles and summaries of the remaining articles, eliminating those that did not meet the inclusion criteria.

All studies considered potentially eligible and classified as relevant were retrieved and evaluated through a full-text review, conducted independently by two reviewers (Y.B.R. and A.V.-S.).

Reading the full text: The reviewers read the full text of the selected articles, and the rejected ones will be resolved by consensus with a third reviewer (J.C.-G). Titles and abstracts of publications identified by the search strategy were screened for subsequent full-text review and cross-checked to identify duplicates.

Likewise, a manual review of the reference lists of all relevant articles was carried out using the “snowball” strategy (Greenhalgh and Peacock, 2005). Based on the information extracted from the full texts, the inclusion and exclusion criteria were systematically applied for the final selection of studies eligible for inclusion in this systematic review. Any discrepancies in the selection process were resolved through discussion and consensus between two reviewers (Y.B.R and A.V.-S).

### 2.4 Data extraction

After applying the inclusion and exclusion criteria to each study, the following data were systematically extracted: source of the study [author(s) and year of publication], sample characteristics, sample size, methods, variables analyzed, results, and observed effects.

For each study included, information from all eligible publications was compiled exhaustively. Mean values (±), standard deviation (SD), and sample size were extracted from the tables of all included articles. Any discrepancies in the data extraction process were resolved through discussion until consensus was reached among the reviewers.

### 2.5 Quality assessment and risk of bias

In accordance with PRISMA guidelines, the methodological quality and risk of bias of the included studies were independently assessed by two reviewers (Y.B.R. and A.V.-S.). In case of disagreement, discrepancies were resolved by consulting additional reviewers (J.C.-G. and J.L.-C.), following the procedures recommended by the Cochrane Collaboration (Higgins, 2011) and the ROBINS-I (Risk of Bias in Non-randomized Interventions) tool guidelines (Sterne et al., 2016).

The following elements were included in the Cochrane Risk of Bias tool and divided into different domains: (I) selection bias domain with the items random sequence generation and allocation concealment, (II) performance bias domain, with the item blinding of participants and staff, (III) detection bias domain, with the item masking of outcome assessment, (IV) attrition bias domain, with the incomplete outcome data item, (V) reporting bias domain, with the selective reporting item, and (VI) other bias domain, with the other bias item.

The criteria were classified for each study as “low” (unlikely to seriously alter the results), ‘high’ (seriously undermining the reliability of the results), and “unclear” (raising doubts about the results).

On the other hand, the Physiotherapy Evidence Database (PEDro) scale was used to analyze methodological quality. This scale assesses the methodological quality of clinical designs (**Table 1**). This tool is based on a checklist developed by Verhagen (Verhagen et al., 1998) using the Delphi technique (Alt Murphy et al., 2015).

**Table 1:** Physiotherapy evidence database (PEDro) scale to analyze the methodological quality of the studies.

PEDro scale			
1	The selection criteria were specified as follows	no	yes
2	Subjects were randomly assigned to groups (in a crossover study, subjects were randomly distributed as they received treatments)	no	yes
3	The assignment was concealed	no	yes
4	The groups were similar at baseline in relation to the most important prognostic indicators	no	yes
5	All subjects were blinded	no	yes
6	All therapists who administered the therapy were blinded	no	yes
7	All assessors that measured at least one key outcome were blinded	no	yes
8	Measures of at least one of the key outcomes were obtained from more than 85% of the subjects initially assigned to the groups	no	yes
9	Results were presented for all subjects who received treatment or were assigned to the control group, or when this could not be done, data for at least one key outcome was analyzed on an “intention-to-treat” basis	no	yes
10	Results of statistical comparisons between groups were reported for at least one key outcome	no	yes
11	The study provides point and variability measures for at least one key outcome	no	yes

The PEDro scale consists of a total of 11 items. Item 1 assesses the external validity of the study, while items 2 to 9 are related to internal validity. Items 10 and 11 determine whether the statistical information reported allows for an adequate and accurate interpretation of the results. Each item is classified dichotomously as “yes,” “no,” or “not reported.” Only affirmative (“yes”) responses receive a score of one point, while ‘no’ or “not reported” responses receive no score.

In this review, item 1 of the PEDro scale was not considered, as it relates to the assessment of external validity. Consequently, only items 2 to 11 were included for the assessment of methodological quality. Therefore, the maximum possible score per study was 10 points, while the minimum score was 0 points.

Based on the results obtained using the Cochrane risk of bias tool and given that most studies had a non-randomized design, the ROBINS-I tool was prioritized using the following seven criteria: (I) bias due to confounding, (II) bias in the selection of participants for the study, (III) bias in the classification of interventions, (IV) bias due to deviations from intended interventions, (V) bias due to missing data, (VI) bias in measurement of outcomes, (VII) bias in selection of the reported result. The criteria were classified for each study as “low risk of bias,” “moderate risk of bias,” “serious risk of bias,” “critical risk of bias,” and “insufficient information”.

To conduct the most comprehensive and specific assessment of methodological quality, ROBINS-I was used as the primary tool, supplemented by the Cochrane risk of bias tool, which confirmed that all studies had a low risk of bias in the following domains: (IV) dropout bias, with the item “incomplete outcome data”; (V) reporting bias domain, with the item “selective reporting”; and (VI) other biases domain, with the item “other biases”.

## 3 Results

### 3.1 Search result

**Figure 2:**
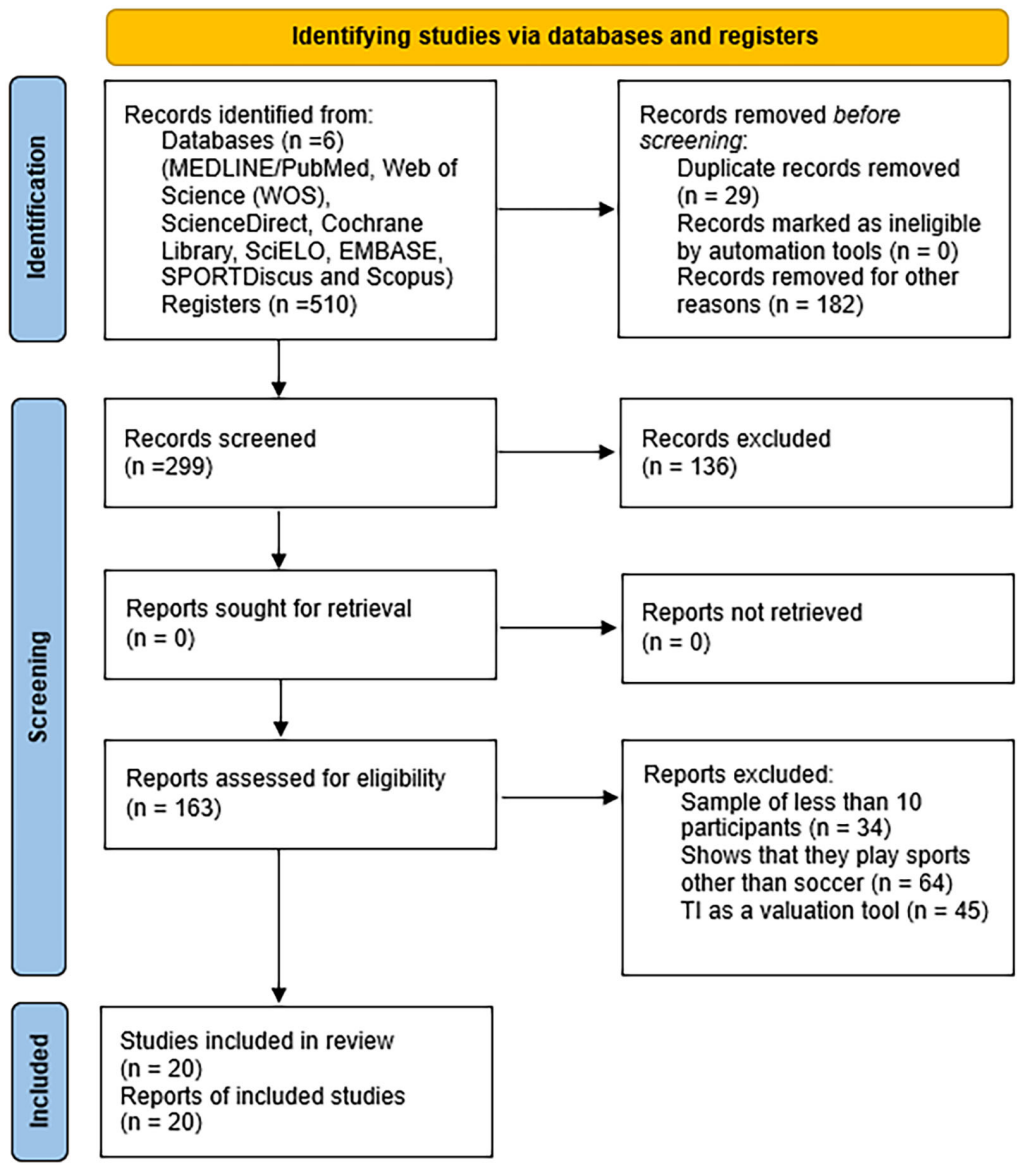
Flow diagram of the study selection.

The database search yielded 510 articles. Subsequently, using the criterion of duplicates, 29 studies were excluded, publications older than 10 years and in a language other than English were identified, leaving 299 records. In a second stage, the following exclusion criteria was applied: Systematic reviews, abstracts of conferences and books, master’s or doctoral theses, finding 136 articles, decreasing the number of studies to 163. As the final screening and analysis stage, relevant criteria specific to the subject and objectives of the study were applied: a sample size equal to or greater than 10 participants, sample characteristics indicating that participants were soccer players, and the use of Infrared Thermography as an evaluation tool. As a result, a total of 20 articles fully met the inclusion and exclusion criteria and were included in this systematic review, as illustrated in the flow chart (**Figure 2**).

A total of 20 studies were included from the final selection. A total of 9 articles were included (Dębiec-Bąk et al., 2016; Rodriguez‐Sanz et al., 2018; Alexander et al., 2021; Alexander et al., 2022; Duarte et al., 2022; Lubkowska and Knyszyńska, 2023; Majano et al., 2023b; de Andrade Fernandes et al., 2024; Fernandes et al., 2025) with significant data concerning IRT for fatigue assessment and personalized recovery, 11 articles (de Andrade Fernandes et al., 2017; Menezes et al., 2018; Côrte et al., 2019; Gomez-Carmona et al., 2020; Mendonça Teixeira et al., 2020; de Carvalho et al., 2021; Júnior et al., 2021; Escamilla-Galindo et al., 2023; Majano et al., 2023b; Majano et al., 2023a; Majano et al., 2024) in the assessment of injury risk, and 2 articles (Duarte et al., 2022; Escamilla-Galindo et al., 2025) on the use of IRT for readaptation. Two articles (Duarte et al., 2022; Majano et al., 2023b), provided information on more than one topic of discussion. As a result, some articles addressed more than one subject area; therefore, the total number of articles across all three categories amounts to twenty-two, exceeding the total number of included studies, which is twenty.

### 3.2 Study characteristics

The source or reference of the study (author/authors and year of publication); sample population; methods describing the technological tools used for the evaluations; study variables, type of research design; main findings and effects of the intervention are shown in **Table 2**.

**Table 2:** Methodology and results of the interventions.

Reference	N	Methods	Variables	Design	Results	Effect
Fernandes et al. (2025)	30	Infrared Thermography. Reflotron CK Reagents.	Skin Temperature. % Creatine kinase (CK)	A prospective longitudinal study	Skin temperature can complement CK measurements.	↑
Escamilla et al (Escamilla-Galindo et al., 2025)	30	Infrared Thermography.	Knee Skin Temperature Asymmetry	Linear mixed model.	The IRT establishes objective criteria for monitoring the stages of rehabilitation in soccer players’ return to play.	↑
Majano et al. (2024)	20	Infrared Thermography. Cryostimulation	Thermal Asymmetry	Experimental study, with repeated measures and longitudinal cut-off	The greatest reduction in thermal asymmetry occurs in the hamstrings.	↑
de Andrade et al (de Andrade Fernandes et al., 2024)	30	Infrared Thermography.	Cold pixels. Neutral pixels. Hot pixels.	Thermopixelography, a prospective longitudinal study,	With IRT, the image analysis process is faster, more accurate, and covers all areas of the muscles.	↑
Majano et al. (2023b)	20	Infrared Themographics. Optojump infrared device. Photocells to determine	Repeated sprint ability test (RSA). Fatigue. Average jump height. Average Temperature Asymmetry.	This study used repeated measures.	Thermography can provide information on CMJ and RSA performance for hamstring asymmetries greater than 0.2 °C.	↑
Majano et al. (2023a)	30	Infrared Themographics. GPS devices. Perceived Well-Being Questionnaire.	Stress, muscle pain, rest time and quality of rest. External Load.Thermal Asymmetries.	This study used repeated measures.	Greater asymmetry leads to poorer well-being among players.	↑
Escamilla et al (Escamilla-Galindo et al., 2023)	31	Infrared Thermography. Isometric Strength Test.	Thermal Asymmetry of the hamstrings. Hamstring Muscle Strength Asymmetry.	Cross-sectional study	There is no direct relationship between isolated isometric strength and temperature asymmetry.	↓
Lubkowska et al (Lubkowska and Knyszyńska, 2023)	14	Infrared Thermography. Partial Body Cryostimulation (PBC)	Skin temperature (Tsk), creatine kinase (CK), lactate dehydrogenase (LDH), aspartate aminotransferase (AST)	This study used within participants repeated measures to determine the relationships between variables.	PBC has a positive influence on post-match recovery, and thermography is a suitable method for estimating temperature changes.	↑
Alexander et al. (2022)	24	Infrared Thermograph, Global Positioning System (GPS), Recovery Tub Solo and Digital Multimeter	Cold water immersion (CWI). Passive recovery (PR). Eccentric strength of the hamstrings. Isometric strength of the adductors. Flexibility of the hamstrings. Skin surface temperature (Tsk).	Longitudinal Study	Significant differences were between CWI and RP hamstring eccentric strength immediately after intervention.	↑
Duarte et al. (2022)	11	Infrared Thermography. Blood Samples.	C Reactive Protein (CRP). Skin temperature.	This study used repeated measures within participants to determine the relationship between C-Reactive Protein (CRP) and Skin Temperature (Tsk) of the lower limbs (LLs).	The 48 hours following a match provide information in terms of the magnitude and duration of the inflammatory processes associated with recovery.	↑
de Carvalho et al. (2021)	22	Infrared Thermography. GPS global positioning system. YOYO IR - Intermittent Recovery Test. CMJ jump. Clinical recovery scales.	Average Aperture Asymmetry. CK. Distances traveled, accelerations, decelerations, intensity, speeds. Perception of recovery, pain and fatigue.	Single-blind cross-sectional study	IRT of the lower limbs of professional soccer players did not correlate with CK level, pain, perceived fatigue or recovery, or on-field performance variables.	↓
Rodrigues et al (Júnior et al., 2021)	20	Infrared Thermography. Blood Samples. CMJ Jumps.	Mucosal damage and inflammatory condition. Strength asymmetry. Thermal Asymmetries.	Multiparametric observational design, composed of two evaluation sessions.	Suggests that the 72-hour interval between competitive seasons is not sufficient for complete recovery.	↑
Alexander et al. (2021)	18	Infrared Thermography. Cryocompression. Yo-Yo fatigue test	Cooling Intervention. Fatigue Protocol. Skin Temperature. Eccentric strength of hamstrings.	Randomized, longitudinal cross-sectional design.	Significant decreases in TSk in the posterior thigh were reported for all time points compared to pre-cryocompression temperatures (p = < 0.05).	↑
Mendonça et al (Mendonça Teixeira et al., 2020)	59	Infrared Thermography. Isokinetic Dynamometer.	Thermal Asymmetry of Quadriceps and Hamstrings. Quadriceps and Hamstring Maximum Torque	Cross-sectional study	Thermal differences between hamstrings and quadriceps could be closely related to thermoregulatory factors than to strength imbalances.	↓
Gomez et al (Gomez-Carmona et al., 2020)	24	Infrared Thermography.	Bilateral thermal asymmetry. Incidence of injury (frequency, location, type and mechanism).	Cross-sectional, prospective study design to compare 2 injury prevention programs.	The use of IRT, ensured that the incidence of injury and days lost due to injury in the season were reduced.	↑
Côrte et al. (2019)	28	Infrared Thermography. Ultrasound examination.	Thermal Asymmetries. Muscle Injury.	A prospective longitudinal study.	Early identification of injury risk, using IRT and the preventive protocol applied, helps minimize recurrence.	↑
Rodriguez et al (Rodriguez‐Sanz et al., 2018)	35	Infrared Thermography.	Opening of the gastrocnemius. Achilles tendon temperature	Cross-sectional study of second level of care.	IRT evaluation of the gastrocnemius and Achilles tendon is suitable for differing equinus gastrocnemius-soleus.	↑
Menezes et al. (2018)	26	Infrared thermography. My Jump. Strength test for bench press, front lateral pulldown, shoulder press, leg press, leg curl and squat.	Asymmetries, high, low and medium temperatures. Vertical jump performance. Maximum Repetition (1RM).	Experimental study, with repeated measures and longitudinal cut-off	Preventive IRT mapping should focus on moderate injuries to the muscles and tendons of the lower extremities, and improve diagnosis and control of injury risk.	↑
Andrade et al (de Andrade Fernandes et al., 2017)	10	Infrared Thermography	Mean lower limb skin temperatures and CK concentration.	Cross-sectional study	The greatest inflammatory response occurs in the second match, which was preceded by only three days of recovery.	↑
Dębiec et al (Dębiec-Bąk et al., 2016)	60	Infrared Thermography. Cryostimulation	Body temperature.	Experimental study, with repeated measures and longitudinal cut-off	The results of the study indicate a greater effectiveness of thermoregulatory processes in soccer players.	↑

↑, Positive effect; ↓, Negative effect; N, Sample or participating subjects; CRP, C-Reactive Protein; CK, Creatine kinase; RSA, Repeated sprint ability test; Tsk, Skin temperature; LDH, lactate dehydrogenase; AST, aspartate aminotransferase; GPS, Global Positioning System; CWI, Cold water immersion; PR, Passive recovery; LLs, lower limbs; 1RM, One Repetition Maximum.

A total of 9 articles (Dębiec-Bąk et al., 2016; Rodriguez‐Sanz et al., 2018; Alexander et al., 2021; Alexander et al., 2022; Duarte et al., 2022; Lubkowska and Knyszyńska, 2023; Majano et al., 2023b; de Andrade Fernandes et al., 2024; Fernandes et al., 2025) with important information on IRT for fatigue assessment, 11 studies (de Andrade Fernandes et al., 2017; Menezes et al., 2018; Côrte et al., 2019; Gomez-Carmona et al., 2020; Mendonça Teixeira et al., 2020; de Carvalho et al., 2021; Júnior et al., 2021; Escamilla-Galindo et al., 2023; Majano et al., 2023b; Majano et al., 2023a; Majano et al., 2024) with guidance on the use of IRT in the assessment of injury risk factors, and 2 studies (Duarte et al., 2022; Escamilla-Galindo et al., 2025) that included infrared thermography in the rehabilitation processes of soccer players, as an important basis for their return to the field of play.

### 3.3 Risk of bias

**Figure 3:**
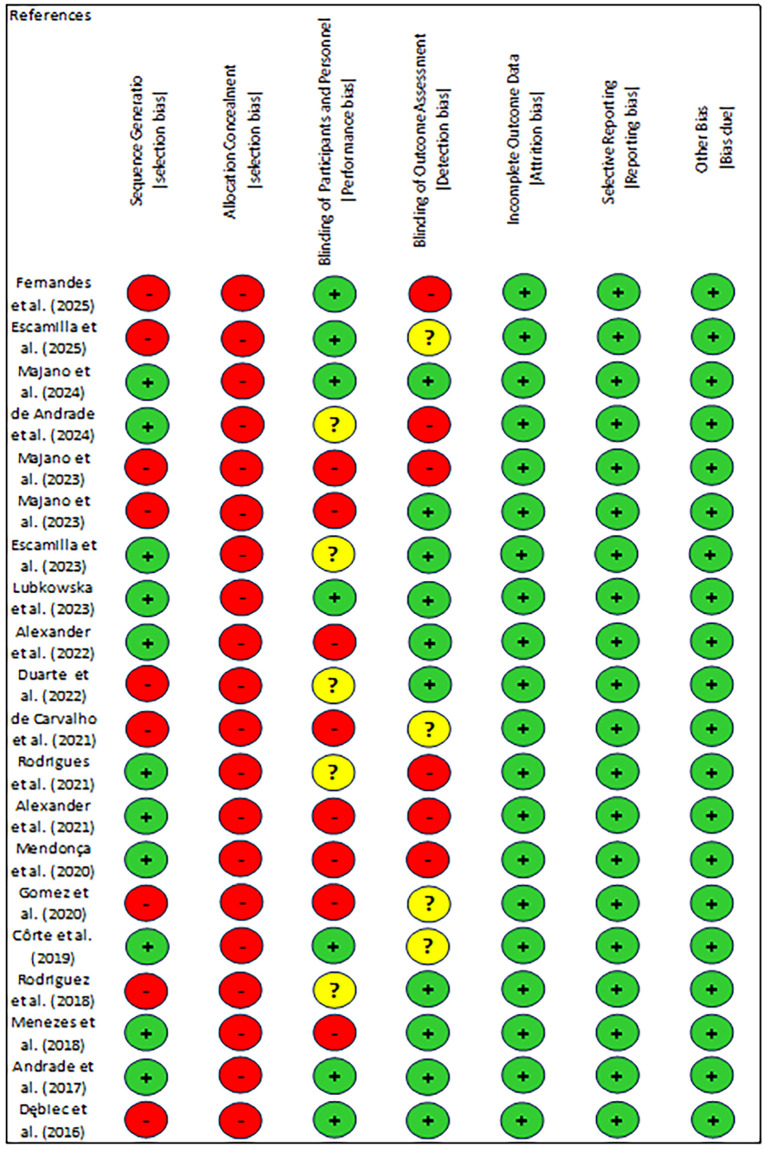
Risk of bias summary: authors’ judgments about each risk of bias item, presented as percentages across all included studies.

**Figure 4:**
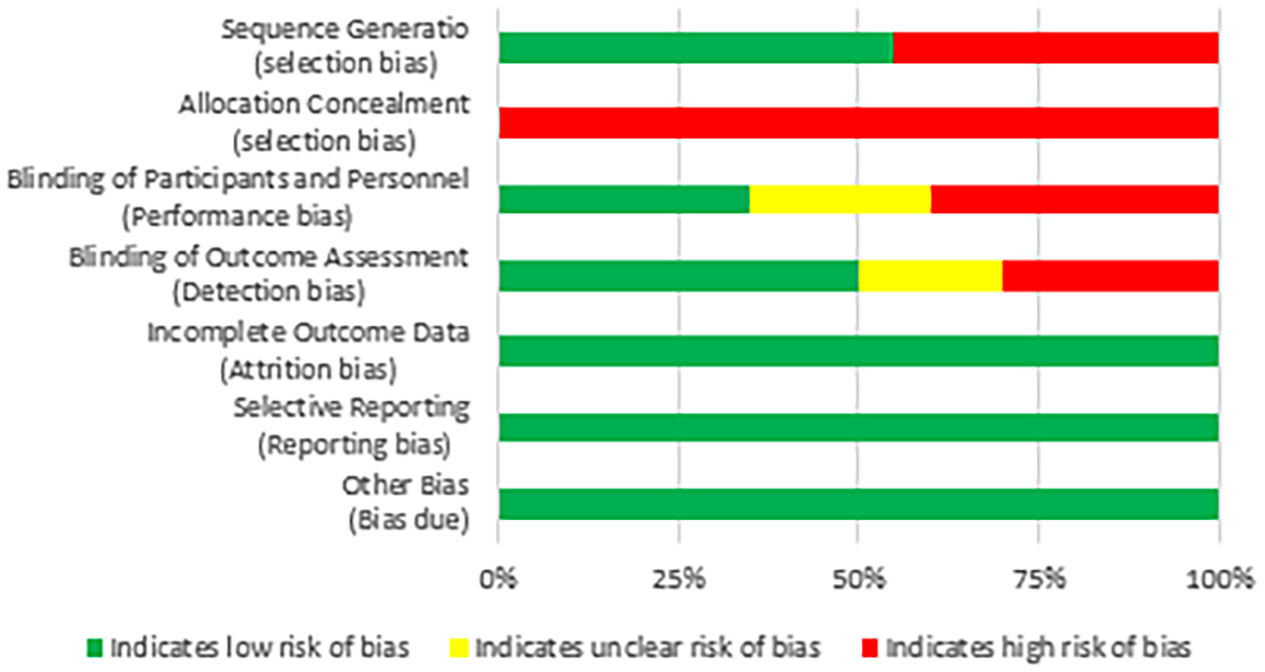
Risk of bias summary: authors’ judgments about each risk of bias item for each included study.

In accordance with the guidelines established by the Cochrane Collaboration (Higgins, 2011) on methodological quality and risk of bias, the studies were evaluated. The complete quality assessments of the study are shown in **Figure 3**. In the risk of bias (**Figure 3**), in the random sequence generation item, the assignment was characterized as high risk to nine studies (Dębiec-Bąk et al., 2016; Rodriguez‐Sanz et al., 2018; Gomez-Carmona et al., 2020; de Carvalho et al., 2021; Duarte et al., 2022; Majano et al., 2023b; Majano et al., 2023a; Escamilla-Galindo et al., 2025; Fernandes et al., 2025) and eleven low-risk studies (de Andrade Fernandes et al., 2017; Menezes et al., 2018; Côrte et al., 2019; Mendonça Teixeira et al., 2020; Alexander et al., 2021; Júnior et al., 2021; Alexander et al., 2022; Escamilla-Galindo et al., 2023; Lubkowska and Knyszyńska, 2023; de Andrade Fernandes et al., 2024; Majano et al., 2024). Regarding the item of allocation concealment, all the studies were categorized as high risk (Dębiec-Bąk et al., 2016; de Andrade Fernandes et al., 2017; Menezes et al., 2018; Rodriguez‐Sanz et al., 2018; Côrte et al., 2019; Gomez-Carmona et al., 2020; Mendonça Teixeira et al., 2020; Alexander et al., 2021; de Carvalho et al., 2021; Júnior et al., 2021; Alexander et al., 2022; Duarte et al., 2022; Escamilla-Galindo et al., 2023; Lubkowska and Knyszyńska, 2023; Majano et al., 2023b; Majano et al., 2023a; de Andrade Fernandes et al., 2024; Majano et al., 2024; Escamilla-Galindo et al., 2025; Fernandes et al., 2025). Regarding the performance bias domain, blinding of participants and personnel, eight studies were determined to be high risk (Menezes et al., 2018; Gomez-Carmona et al., 2020; Mendonça Teixeira et al., 2020; Alexander et al., 2021; de Carvalho et al., 2021; Alexander et al., 2022; Majano et al., 2023b; Majano et al., 2023a) seven studies were found to be low risk (Dębiec-Bąk et al., 2016; de Andrade Fernandes et al., 2017; Côrte et al., 2019; Lubkowska and Knyszyńska, 2023; Majano et al., 2024; Escamilla-Galindo et al., 2025; Fernandes et al., 2025) and five studies were classified in the unclear risk of bias category (Rodriguez‐Sanz et al., 2018; Júnior et al., 2021; Duarte et al., 2022; Escamilla-Galindo et al., 2023; de Andrade Fernandes et al., 2024). Detection bias with its item masking of outcome assessment, ten studies were categorized as at low risk of bias (Dębiec-Bąk et al., 2016; de Andrade Fernandes et al., 2017; Menezes et al., 2018; Rodriguez‐Sanz et al., 2018; Alexander et al., 2022; Duarte et al., 2022; Escamilla-Galindo et al., 2023; Lubkowska and Knyszyńska, 2023; Majano et al., 2023a; Majano et al., 2024) six studies were considered high risk (Mendonça Teixeira et al., 2020; Alexander et al., 2021; Júnior et al., 2021; Majano et al., 2023b; de Andrade Fernandes et al., 2024; Fernandes et al., 2025) and in the unclear risk of bias category, four studies were found (Côrte et al., 2019; Gomez-Carmona et al., 2020; de Carvalho et al., 2021; Escamilla-Galindo et al., 2025). Regarding the domains of attrition bias, reporting bias and bias due to other problems with the item’s incomplete outcome data, selective reporting and other biases respectively, all twenty studies were considered with a low risk of bias category (Dębiec-Bąk et al., 2016; de Andrade Fernandes et al., 2017; Menezes et al., 2018; Rodriguez‐Sanz et al., 2018; Côrte et al., 2019; Gomez-Carmona et al., 2020; Mendonça Teixeira et al., 2020; Alexander et al., 2021; de Carvalho et al., 2021; Júnior et al., 2021; Alexander et al., 2022; Duarte et al., 2022; Escamilla-Galindo et al., 2023; Lubkowska and Knyszyńska, 2023; Majano et al., 2023b; Majano et al., 2023a; de Andrade Fernandes et al., 2024; Majano et al., 2024; Escamilla-Galindo et al., 2025; Fernandes et al., 2025) (**Figures 3**, **4**).

### 3.4 Methodological quality assessment

The methodological quality of the research included and analyzed ranged between 5 and 9 points, with an average of 6.75/10 points, distributed with 5 points 3 articles, 6 points 8 articles, 7 points 4 articles, 8 points 2 articles and ending with 9 points 3 articles.

Taking into account the differences in the scoring of certain items, there was a notable consistency in the criteria that were clearly met, such as item 4: “the groups were similar at baseline in relation to the most important prognostic indicators”, item 8: “measures of at least one of the key outcomes were obtained from more than 85% of the subjects initially assigned to the groups”, item 9: “results were presented for all subjects who received treatment or were assigned to the control group, or when this could not be done, data for at least one key outcome were analyzed on an intention-to-treat basis”, item 10: “results of statistical comparisons between groups were reported for at least one key outcome” and item 11: “the study provides point and variability measures for at least one key outcome”. On the other hand, no study met criterion 3 “allocation was concealed”. Lastly, 3 studies or investigations met the criteria in their entirety, missing only item 3 (**Table 3**).

**Table 3:** Results according to the PEDro scale (n = 20).

Clinical trial	1	2	3	4	5	6	7	8	9	10	11	Total
Fernandes et al. (2025)	Yes	No	No	Yes	Yes	Yes	No	Yes	Yes	Yes	Yes	7
Escamilla et al (Escamilla-Galindo et al., 2025)	Yes	No	No	Yes	Yes	Yes	No	Yes	Yes	Yes	Yes	7
Majano et al. (2024)	Yes	Yes	No	Yes	Yes	Yes	Yes	Yes	Yes	Yes	Yes	9
de Andrade et al (de Andrade Fernandes et al., 2024)	Yes	Yes	No	Yes	No	No	No	Yes	Yes	Yes	Yes	6
Majano et al. (2023b)	Yes	No	No	Yes	No	No	No	Yes	Yes	Yes	Yes	5
Majano et al. (2023a)	Yes	No	No	Yes	No	No	Yes	Yes	Yes	Yes	Yes	6
Escamilla et al (Escamilla-Galindo et al., 2023)	Yes	Yes	No	Yes	No	No	Yes	Yes	Yes	Yes	Yes	7
Lubkowska et al (Lubkowska and Knyszyńska, 2023)	Yes	Yes	No	Yes	Yes	Yes	Yes	Yes	Yes	Yes	Yes	9
Alexander et al. (2022)	Yes	Yes	No	Yes	No	No	Yes	Yes	Yes	Yes	Yes	7
Duarte et al. (2022)	Yes	No	No	Yes	No	No	Yes	Yes	Yes	Yes	Yes	6
de Carvalho et al. (2021)	Yes	No	No	Yes	No	No	No	Yes	Yes	Yes	Yes	5
Rodrigues et al (Júnior et al., 2021)	Yes	Yes	No	Yes	No	No	No	Yes	Yes	Yes	Yes	6
Alexander et al. (2021)	Yes	Yes	No	Yes	No	No	No	Yes	Yes	Yes	Yes	6
Mendonça et al (Mendonça Teixeira et al., 2020)	Yes	Yes	No	Yes	No	No	No	Yes	Yes	Yes	Yes	6
Gomez et al (Gomez-Carmona et al., 2020)	Yes	No	No	Yes	No	No	No	Yes	Yes	Yes	Yes	5
Côrte et al. (2019)	Yes	Yes	No	Yes	Yes	Yes	No	Yes	Yes	Yes	Yes	8
Rodriguez et al (Rodriguez‐Sanz et al., 2018)	Yes	No	No	Yes	No	No	Yes	Yes	Yes	Yes	Yes	6
Menezes et al. (2018)	Yes	Yes	No	Yes	No	No	Yes	Yes	Yes	Yes	Yes	6
Andrade et al (de Andrade Fernandes et al., 2017)	Yes	Yes	No	Yes	Yes	Yes	Yes	Yes	Yes	Yes	Yes	9
Dębiec et al (Dębiec-Bąk et al., 2016)	Yes	No	No	Yes	Yes	Yes	Yes	Yes	Yes	Yes	Yes	8

**Figure 5:**
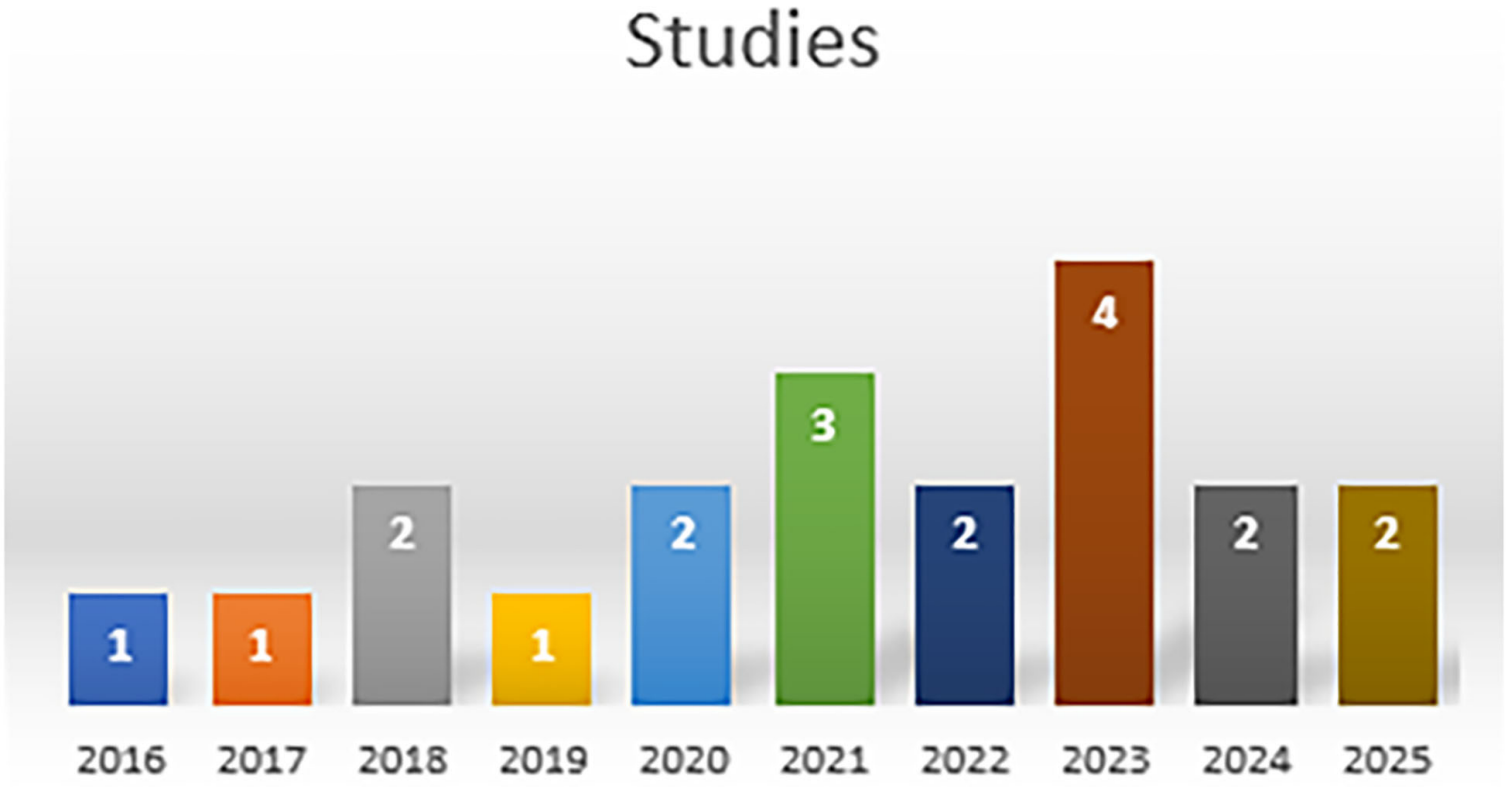
Chronology of the number of published studies related to the use of IRT.

With regard to the chronology of the articles studied (**Figure 5**), it is important to note that, of these twenty studies, 13 have been published in the last 5 years 2 in 2025 (Escamilla-Galindo et al., 2025; Fernandes et al., 2025) 2 in 2024 (de Andrade Fernandes et al., 2024; Majano et al., 2024) 4 in 2023 (Escamilla-Galindo et al., 2023; Lubkowska and Knyszyńska, 2023; Majano et al., 2023b; Majano et al., 2023a) 2 in 2022 (Alexander et al., 2022; Duarte et al., 2022) and 3 in 2021 (Alexander et al., 2021; de Carvalho et al., 2021; Júnior et al., 2021). The remaining research corresponds to 7 studies, which are divided into 2 in 2020 (Gomez-Carmona et al., 2020; Mendonça Teixeira et al., 2020) 1 in 2019 (Côrte et al., 2019) 2 in 2018 (Menezes et al., 2018; Rodriguez‐Sanz et al., 2018); 1 in 2017 (de Andrade Fernandes et al., 2017) and lastly 1 in 2016 (Dębiec-Bąk et al., 2016). The above shows the great interest and importance in sports science, the use of IRT as a technological tool in the processes of sports performance.

Firstly, 9 studies concluded that IRT is a valuable tool for detecting fatigue through thermal asymmetries (Rodriguez‐Sanz et al., 2018; Majano et al., 2023b; de Andrade Fernandes et al., 2024), creating an evaluation protocol with the characteristics of establishing a baseline a day before the competition (M1), 24 hours after (M2), 48 hours after (M3), and 72 hours after (M4), arguing that 48 hours of rest is not enough for the complete recovery of an athlete (**Table 4**).

**Table 4:** Summary of the use of IRT to assess fatigue, injury risk factors and recovery.

N° studies	Thematic	Conclusion
9	IRT for fatigue assessment and personalized recovery	48 hours of rest may not be enough for an athlete’s full recovery
11	IRT for injury risk assessment	Hyperthermia or muscular hypothermia should be considered as a factor in assessing the risk of injury
2	IRT for the readaptation process	Prolonging a player’s return to participating in sports, until the stability of thermal asymmetries is reached, favoring the optimal state of fitness

**Figure 6:**
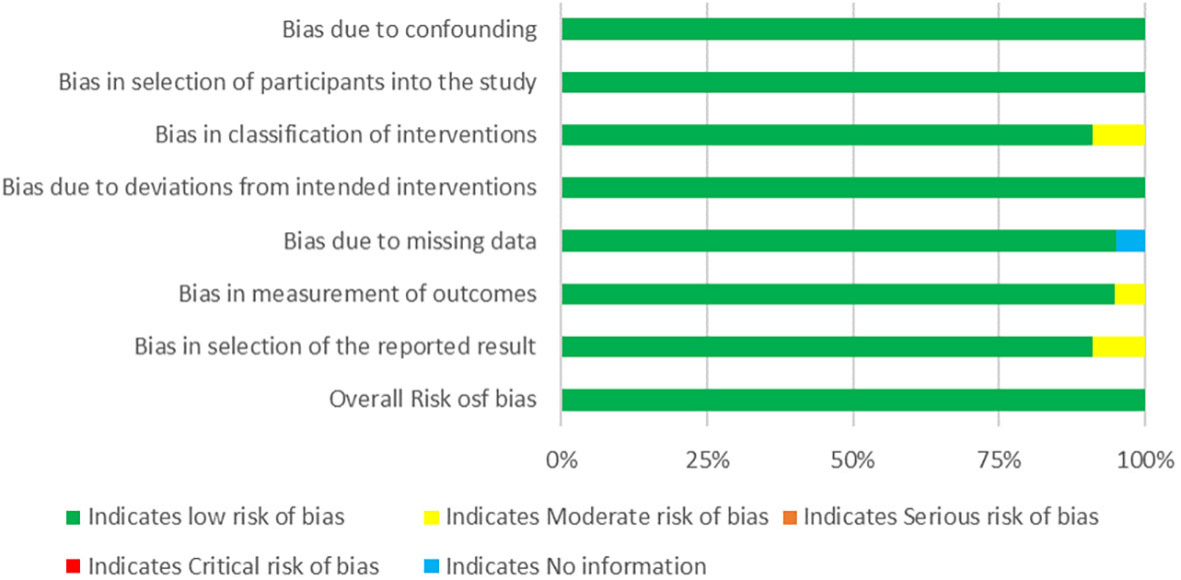
Summary of the ROBINS-I risk of bias: authors’ assessments of each risk of bias factor, expressed as percentages for the pooled set of included studies.

**Figure 7:**
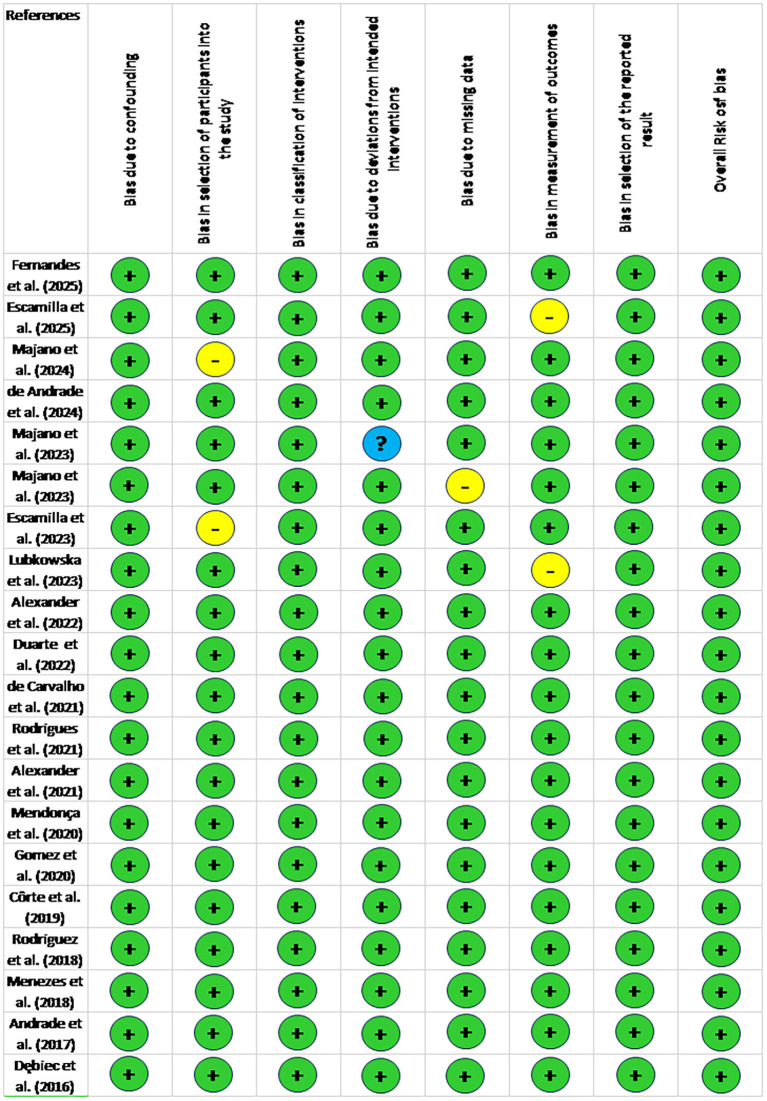
Summary of the ROBINS-I risk of bias, authors’ assessments of each risk of bias factor for each included study.

Given the above results and considering that all studies have a “high risk” of allocation concealment and are predominantly non-randomized intervention studies, a risk of bias assessment was conducted using the ROBINS-I (Risk of Bias in Non-randomized Interventions) tool guidelines (Sterne et al., 2016). Across the seven criteria for assessing risk of bias, no results indicating serious or critical risk of bias were found; instead, moderate risk of bias predominated in the studies, as shown in **Figures 6**, **7**. Specifically, in the Pre-intervention domain, the criterion “bias in study participant selection” is characterized by a “moderate risk of bias,” indicating that it is robust only for non-randomized studies; and the criterion “bias due to confounding factors” this is determined by five studies (de Andrade Fernandes et al., 2017; Côrte et al., 2019; Alexander et al., 2021; Alexander et al., 2022; de Andrade Fernandes et al., 2024) with a low risk of bias and the remaining fifteen one with a moderate risk of bias; for the criterion “bias in the classification of interventions,” four studies (de Andrade Fernandes et al., 2017; Côrte et al., 2019; Lubkowska and Knyszyńska, 2023; Escamilla-Galindo et al., 2025) have a low risk of bias assessment and sixteen have a moderate risk of bias; for the criterion “bias due to deviations from planned interventions,” one study (Majano et al., 2023b) did not provide information on which to base a judgment regarding the risk of bias; for the item “bias due to missing data,” one study (Majano et al., 2023a) is characterized as having a “moderate risk of bias”; and, finally, two studies (Lubkowska and Knyszyńska, 2023; Escamilla-Galindo et al., 2025), under the criterion of “bias in outcome measurement,” present a “moderate risk of bias.” The overall risk of bias for the studies included in this review is classified as “moderate risk of bias,” which is generally consistent for non-randomized studies.

## 4 Discussion

### 4.1 Summary of the main results

This systematic review compiled, analyzed, and consolidated the most recent evidence from published international research on the use of infrared thermography to assess fatigue, injury risk factors, and recovery in soccer players. A literature review was conducted, including the most relevant and influential published studies, in order to gather the key findings cited by their authors. This approach aimed to address all critical aspects to consider during and after sports practice, with the objective of detecting fatigue and risk factors for injury.

The main findings suggest that IRT is a promising tool for detecting fatigue through thermal asymmetries and for assessing injury risk. However, the methodological quality of the primary studies presents limitations that should be considered when interpreting these results.

Infrared thermography (IRT) as the primary tool, the method proved to be effective and reliable. The identification of muscle fatigue, the risk of injury and the recovery of the athlete are considered key and fundamental aspects for two main objectives related to this topic: detecting changes in skin temperature over time during recovery, and evaluating the effectiveness and efficiency of recovery strategies and protocols. However, concerning the recovery of soccer players using protocols and methods based on cryostimulation determining physiological and biomechanical responses through IRT, the available evidence remains limited for the best of the authors knowledge. The physiological interpretation of the mechanisms, with the relationship between skin temperature and structural vs. metabolic fatigue, is presented theoretically but without being contrasted with solid physiological evidence.

**Figure 8:**
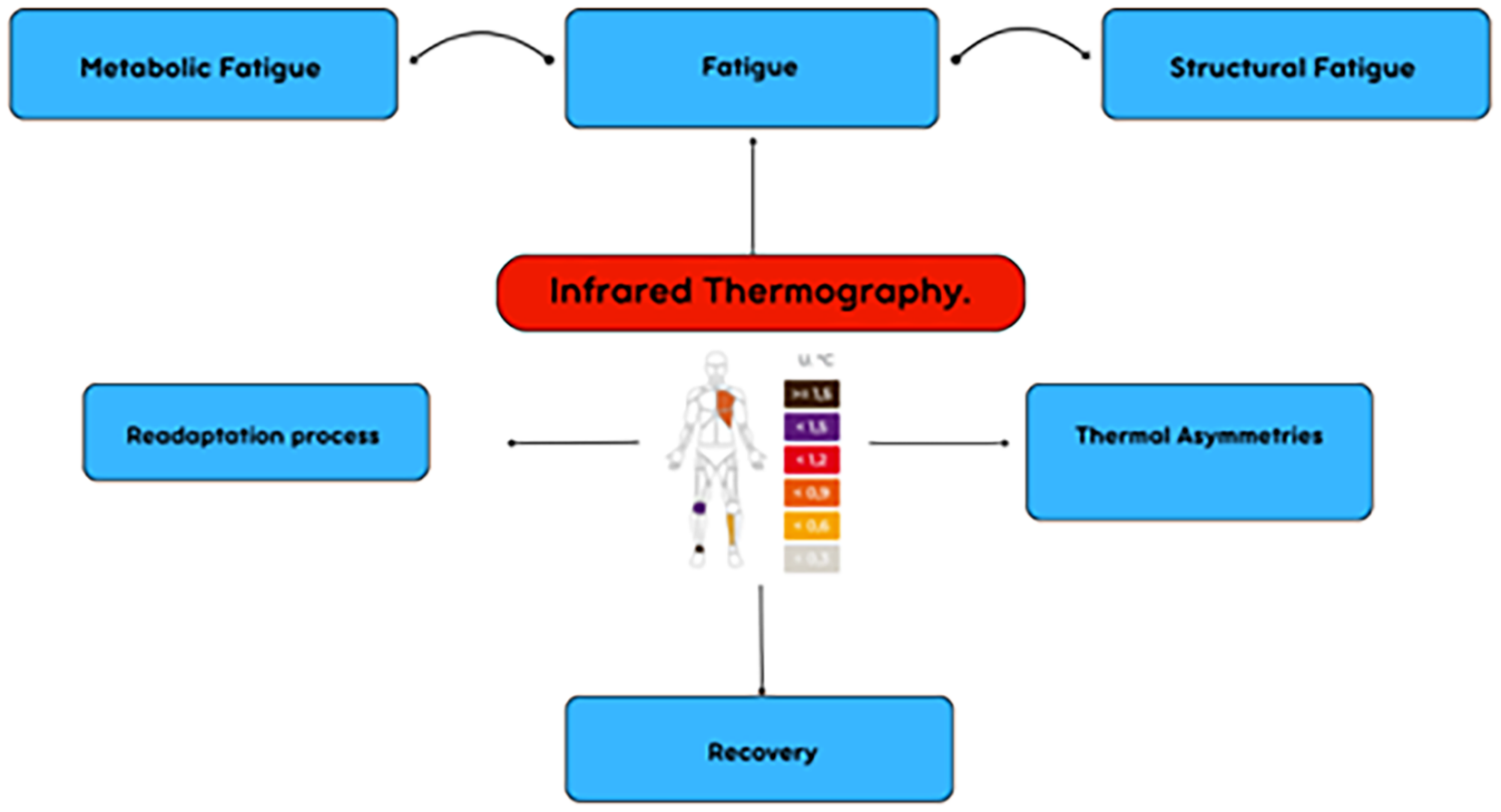
Application of infrared thermography in soccer.

Thus, this review highlights the importance of using IRT in three key areas, which are: IRT for fatigue assessment and personalized recovery, IRT for injury risk assessment, and IRT for the readaptation process (**Figure 8**).

### 4.2 Infrared thermography for fatigue assessment and personalized recovery

The physical demands placed on soccer players, due to the nature of the sport, which involves constant movement, changes in speed, explosive actions, and intermittent efforts, require athletes to perform at maximum intensity. In that sense, the IRT has proven to be a valuable tool for detecting fatigue through thermal asymmetries (Rodriguez‐Sanz et al., 2018; Majano et al., 2023b; de Andrade Fernandes et al., 2024). For this reason, it is advisable to develop evaluation and monitoring protocols using IRT, establishing a baseline before a competition (M1), 24 hours after (M2) and 48 hours after a competition (M3) (Duarte et al., 2022; Majano et al., 2023b; Fernandes et al., 2025) the researchers were able to establish that the skin temperature values of soccer players after 48 hours of a competition (M3) did not return to their initial baseline values (Duarte et al., 2022; Majano et al., 2023b; Fernandes et al., 2025), underlining that 48 hours of rest may not be sufficient for the athlete’s complete recovery. Short rest intervals among competitions directly influence physiological stress and a cumulative problem of loads, directly affecting players displaying fatigue and inadequate recovery processes (Duarte et al., 2022; Majano et al., 2023b).

On the other hand, fatigue being a factor of non-contact injury in soccer, significantly influences sports performance, determining that eccentric training of the muscle and increasing its resistance to load greatly helps in reducing the incidence of injuries (Alexander et al., 2021).

Delayed muscle soreness, up to 72 hours after competition, is considered a primary factor in the inflection of muscle function (Alexander et al., 2021). Therefore, the recovery processes in soccer through cryotherapy monitored by IRT becomes highly relevant, significantly reducing fatigue symptoms (Dębiec-Bąk et al., 2016; Alexander et al., 2021; Alexander et al., 2022; Lubkowska and Knyszyńska, 2023). Cryostimulation studies define temperature control through IRT as an objective, safe and efficient parameter in the diagnosis of this recovery process (Lubkowska and Knyszyńska, 2023). This is based on the fact that the main thermoregulatory organ is the skin (Alexander et al., 2021; Alexander et al., 2022; Lubkowska and Knyszyńska, 2023), and it highlights the existing relationship between the skin and the sympathetic nervous activity of the vasoconstrictive skin and the central temperature (Lubkowska and Knyszyńska, 2023). Besides, skin temperature changes influence thermogenesis; when skin temperature decreases, peripheral sensors are activated, increasing the metabolic response (Alexander et al., 2022; Lubkowska and Knyszyńska, 2023). Furthermore, it is easier to identify skin temperature than intramuscular temperature (Dębiec-Bąk et al., 2016; Alexander et al., 2021; Alexander et al., 2022; Lubkowska and Knyszyńska, 2023).

### 4.3 Infrared thermography for injury risk assessment

Thermal asymmetry is a crucial variable when evaluating the risk of injury (de Andrade Fernandes et al., 2017; Gomez-Carmona et al., 2020; Escamilla-Galindo et al., 2023; Majano et al., 2023b; Majano et al., 2023a; Majano et al., 2024) along with physiological changes, biochemical markers, performance data during official matches, strength asymmetry, external load, vertical jump, creatine kinase (CK) and physical demands (de Andrade Fernandes et al., 2017; Menezes et al., 2018; Mendonça Teixeira et al., 2020; de Carvalho et al., 2021; Júnior et al., 2021; Escamilla-Galindo et al., 2023; Majano et al., 2023b; Majano et al., 2023a; Fernandes et al., 2025).

Sports competitions with extremely short intervals associated with training, travel, and climate changes disrupt athletes’ rest and sleep, which impairs player recovery, increasing the risk factors for injury (Majano et al., 2023a). Studies suggest a response window of delayed muscle soreness, increasing CK and IRT application, within 48 to 72 hours post competition (de Andrade Fernandes et al., 2017; de Carvalho et al., 2021) with the aim of identifying factors that register risk of injury. The homeostatic relationship with body temperature in healthy athletes is a value of asymmetry of less than 0.3°C (Majano et al., 2023a). Therefore, if hyperthermia or hypothermia occurs, these results must be considered and the risk of injury must be assessed (Majano et al., 2023b; Majano et al., 2023a). Given this, it is important to note that for elite soccer players, high-intensity actions influence the development of lower limb asymmetries, specifically in the hamstrings, adductors (Majano et al., 2023a), and quadriceps (Majano et al., 2023b), limiting the performance of demanding tasks in high-level competition and resulting in longer recovery times for both asymmetries and adductor pain (Majano et al., 2023a).

On the other hand, studies (Mendonça Teixeira et al., 2020; de Carvalho et al., 2021; Escamilla-Galindo et al., 2023) demonstrate the importance of establishing a more effective evaluation window, with the aim of correlating or supplementing information on variables such as lower limb pain, elevated CK levels, feelings of fatigue, and GPS data, bearing in mind that a single tool, in this case the IRT, is not sufficient to determine injury risk factors, much less replace other evaluations (de Carvalho et al., 2021) or assessments of soccer players; rather, it should serve as a scientifically sound complement to others, helping to establish conclusions and facilitate timely and rapid decision-making.

According to studies, 92% of injuries in soccer players (Côrte et al., 2019; Gomez-Carmona et al., 2020) are centered in the lower extremities, distributed as 37% in hamstrings, 23% adductors, 19% quadriceps and 13% calf, with hamstring injury being the most common. A professional soccer team with 25 players may typically have a statistic of 15 muscle injuries per season, which would lead to a 2-week injury absence for a player (Côrte et al., 2019). This is a devastating loss of time for a player and their team that will result in poor individual and team performance. Therefore, the implementation of IRT protocols for evaluation, monitoring, and control could be essential. Objective reports and the communication of skin temperature changes in players’ regions of interest (ROI) to the coaching and medical staff has been shown to significantly reduce injury risk, increase player availability, and enhance overall sports performance (Gomez-Carmona et al., 2020).

### 4.4 Infrared thermography for the readaptation process

The planning of readiness and return to play after a sports injury is one of the most critical and concerning moments for athletes who belong to professional soccer clubs. In addition to fatigue evaluation, studies (Escamilla-Galindo et al., 2025) highlight the importance of IRT in the return-to-play process, as its objective, precise and clear characteristics of IRT make it possible to establish physiological criteria for monitoring the state of the ROI in the different phases of rehabilitation and return to competition in soccer players (Escamilla-Galindo et al., 2025). There is a progressive tendency to reduce asymmetry and temperature until the end of the recovery processes, with IRT playing fundamental role in physiological changes from stress to recovery, with the aim of preventing possible recurrences and ensuring a safe return to play (Escamilla-Galindo et al., 2025). Variables, such as the number of training sessions, travel, changes in weather and schedules, competitions with short rest intervals, etc., are all factors that affect the quality of training (Duarte et al., 2022) and negatively affect sports performance (Duarte et al., 2022). These factors may disrupt the stimulus-response relationship and contribute to a higher risk of reinjury in soccer players (Duarte et al., 2022; Escamilla-Galindo et al., 2025). In view of the above, some studies (Escamilla-Galindo et al., 2025) highlight the importance of prolonging the return-to-play for players to favor optimal readiness (Duarte et al., 2022). The gradual stabilization of thermal asymmetries following surgery will allow for an objective and progressive delineation of the different phases of readaptation and return to competition.

## 5 Strengths, limitations and future lines of research

This systematic review of original researches on the use of infrared thermography in soccer highlights the importance of evaluation protocols to identify the different risk factors for injury and implement a monitoring protocol and individualized recovery programs.

Most studies consistently met criteria related to internal validity and data presentation, such as similarity of groups at baseline (item 4), follow-up of more than 85% of subjects (item 8), intention-to-treat analysis (item 9), and presentation of statistical comparisons (items 10 and 11).

Using the ROBINS-I tool, a moderate risk of bias was found to predominate across the studies, with 100% of them showing a moderate risk of bias in participant selection—a robust result for non-randomized studies. Similarly, 75% were rated as robust regarding confounding factors and the classification of interventions; on the other hand, 100% of the studies were rated as low risk with respect to the selection of reported outcomes.

As with similar studies, the technological evaluation tool, the sport discipline, and the terminology, may present limitations. This is especially true considering that including certain studies often entails excluding others that might also offer relevant and significant insights. However, the inclusion criterion accounting for both the female and male athletes broadens the scope of the studies, generating results that benefit the entire soccer-playing population.

The conclusions drawn presented in this review are based solely on the articles selected for this study, according to established search strategy, methodology and the eligibility criteria.

A significant limitation of the studies included in this review was the lack of blinding of assessors and, more importantly, allocation concealment. The lack of blinding may introduce a risk of selection bias, as knowledge of the group to which a participant is assigned may influence the interpretation of the results and the internal validity of the study. This finding underscores the need to improve methodological quality in future research.

On the other hand, the specific limitations of IRT are evident in its use, which can be classified into three fundamental aspects: environmental, individual, and technical factors (Fernández-Cuevas et al., 2015). Each of these aspects involves a large number of variables, making it impossible to control them all effectively, which is a weakness. This leads to the development of rigorous protocols tailored to the context in order to reduce the margin of error in IRT assessments (Marins et al., 2013).

Future research may further explore the effectiveness of infrared thermography in injury rehabilitation protocols within soccer. Other relevant areas of interest include identifying fatigue at different times of the season in soccer, and classifying the thermal profile of players with respect to their position on the field. These studies will solidify and strengthen the use of infrared thermography as a reliable tool in the evaluation, control and monitoring of fatigue and detecting the risk of injury in soccer, for personalized recovery. Beside future research focus on developing standardized thermography protocols, using more rigorous methodological designs (such as allocation concealment), and exploring the use of IRT in different athletic populations to validate its applicability, in lines of research such as the use of more diverse samples, detailed environmental control, longitudinal, multicenter studies, the integration of information technologies with other markers, analysis by position on the field, and sports category.

## 6 Practical applications

Due to the specific characteristics of soccer players and the demands of the sport, the identifying injury risk factors and designing recovery strategies should rely on objective, clear, accurate assessments in the shortest possible time.

Therefore, the importance of employing valid and reliable technologies for evaluation is emphasized.

Our results suggest that teams and coaches should consider the need for longer recovery protocols, where 48 hours of rest may not be sufficient for full recovery, using IRT as an objective monitoring tool to determine players’ physical readiness before returning to competition.

IRT can be used to assess skin temperature, thermal asymmetries in the ROI and structural or metabolic fatigue in order to monitor the risk of injury in soccer players and tailoring recovery programs, collecting data before competition, and again at 24-, 48- and 72-hours post-competition.

Therefore, this systematic review can assist soccer coaches and sports scientists to better understand the use of IRT in fatigue, injury risk and recovery processes in soccer World.

## 7 Conclusions

The results of this systematic review of different studies provide evidence on the use of infrared thermography to assess fatigue, injury risk factors, and monitor the rehabilitation process.

Infrared thermography shows promise as a valid, reliable, and non-invasive tool for assessing skin temperature, reflecting temperature changes in response to physiological processes. It allows for the analysis of structural or metabolic fatigue and thermal asymmetries. Therefore, thermography could be used to design individualized recovery protocols.
